# Health inequalities among children and adolescents in Germany. Developments over time and trends from the KiGGS study

**DOI:** 10.25646/5871

**Published:** 2019-03-14

**Authors:** Thomas Lampert, Jens Hoebel, Benjamin Kuntz, Jonas D. Finger, Heike Hölling, Michael Lange, Elvira Mauz, Gert B. M. Mensink, Christina Poethko-Müller, Anja Schienkiewitz, Anne Starker, Johannes Zeiher, Bärbel-Maria Kurth

**Affiliations:** Robert Koch Institute, Berlin Department of Epidemiology and Health Monitoring

**Keywords:** CHILD AND ADOLESCENT HEALTH, SOCIOECONOMIC STATUS, HEALTH INEQUALITIES, TREND ANALYSES

## Abstract

This study examines the extent to which health inequalities among children and adolescents in Germany have developed over the past decade. The analyses are based on data from the German Health Interview and Examination Survey for Children and Adolescents (KiGGS), which are representative of the 0- to 17-year-old population in Germany. The KiGGS data were collected in three waves: the KiGGS baseline study (2003-2006), KiGGS Wave 1 (2009-2012) and KiGGS Wave 2 (2014-2017). Prevalences of five health outcomes are considered: general health, mental health problems, physical activity, the consumption of sugary soft drinks, and smoking. Moreover, it defines health inequalities in relation to differences in the socioeconomic status of the family (SES), an index derived from the parents’ level of education, occupation and income, and considers both absolute and relative health inequalities. In order to do so, the Slope Index of Inequality (SII) and the Relative Index of Inequality (RII) were calculated using linear probability or log-binomial models. Significant inequalities were identified to the detriment of young people from families with a low SES. These inequalities were particularly pronounced in the KiGGS Wave 2 data with regard to general health and the consumption of sugary soft drinks. Additionally, evidence from trend analyses for these two outcomes suggests that relative inequalities have increased. However, absolute inequalities decreased during the same period, and this also applies to smoking. The persistently high and, in some cases, widened levels of health inequalities indicate that adolescents from families with a low SES do not benefit to the same extent from disease prevention and health promotion measures for children and adolescents as young people from families with a higher SES.

## 1. Introduction

The vast majority of children and adolescents in Germany grow up healthy. Although acute diseases such as upper respiratory tract infections and diarrhoeal diseases are common, these can usually be treated successfully and can even be partly prevented through vaccination [1-3]. Chronic diseases and functional limitations are much less common during childhood and adolescence than in later life. Allergic diseases, however, are widespread among children and adolescents [4-6]. In addition, developmental delays and disorders, e.g. related to motor, psychosocial, and cognitive development, and mental health problems and disorders, such as attention deficit/hyperactivity disorder (ADHD), eating disorders and anxiety disorders, need to be taken into account [7-10].

**The KiGGS study**

The German Health Interview and Examination Survey for Children and Adolescents

**Data owner:** Robert Koch Institute

**Aim:** Providing reliable information on health status, health-related behaviour, living conditions, protective and risk factors, and health care among children, adolescents and young adults living in Germany, with the possibility of trend and longitudinal analyses

**Study design**: Combined cross-sectional and cohort study

**KiGGS survey waves**

► KiGGS baseline study (2003-2006), examination and interview survey
► KiGGS Wave 1 (2009-2012), interview survey
► KiGGS Wave 2 (2014-2017), examination and interview survey

**KiGGS cross-sectional study**

**Population:** Children and adolescents with permanent residence in Germany

**Age range:** 0-17 years

**KiGGS cohort study**

**Sampling:** Re-invitation of everyone who took part in the KiGGS baseline study (n=17,641) and who was willing to participate in a follow-up

**Age range KiGGS Wave 1:** 6-24 years (n=11,992)

**Age range KiGGS Wave 2:** 10-31 years (n=10,853)

More information is available at www.kiggs-studie.de/english

Health development in childhood and adolescence considerably influences people’s health-related opportunities in later life [11, 12]. Studies that demonstrate the association between pre- and perinatal risk factors such as maternal smoking in pregnancy and diseases in later life clearly show that the foundations for health-related behaviour are set early on [13-15]. Empirical evidence also points to a correlation between low birth weight and a risk of cardiovascular disease and type 2 diabetes mellitus in middle age and among the elderly [16, 17]. Additional risk factors in childhood and adolescence have also been shown to increase the long-term risk of chronic disease and disorders; these factors include preterm birth, environmental pollution, exposure to violence [18, 19], an unhealthy diet, unhealthy patterns of exercise, overweight and obesity [20], as well as the consumption of psychoactive substances such as tobacco and alcohol at a young age [21].

The current literature indicates that children and adolescents from socioeconomically disadvantaged families are significantly more likely to face health disadvantages than their peers from families in a better socioeconomic situation [22-26]. These are less pronounced with regard to physical health and infectious diseases, but are particularly evident in the case of developmental disorders in early childhood [27], as well as in mental and psychosocial health [28, 29]. Significant socioeconomic inequalities have also been observed for health-related behaviour and behavioural risk factors such as diet, physical activity and obesity, and for the perinatal risk factors mentioned above [30, 31].

Efforts to improve the health status of children and adolescents have increased in recent years through disease prevention, health promotion measures and health care provision. Children and young people from socioeconomically disadvantaged families are a special target group, not merely because they have greater needs for support and care, but also because existing services fail to reach them in the same way as their peers from families in better social positions [32]. Data that enable a wide-ranging description and analysis of the health situation and unequal socioeconomic distribution of health-related opportunities among children and adolescents are therefore essential for the planning, implementation and evaluation of measures and programmes aimed at promoting child and adolescent health. The Robert Koch Institute’s German Health Interview and Examination Survey for Children and Adolescents (KiGGS) provides an important contribution to making this possible, as, in contrast to many other studies, it covers the majority of relevant health-related areas for the entire period ranging from childhood to adolescence. Moreover, as data is available from the first survey wave, which took place between 2003 and 2006 (KiGGS baseline study), the first follow-up survey, which was conducted between 2009 and 2012 (KiGGS Wave 1), and the latest wave, undertaken between 2014 and 2017 (KiGGS Wave 2), KiGGS can be used to analyse temporal developments and trends over the last ten years.

This work uses data from the KiGGS study to investigate the following three questions, based on selected indicators, about child and adolescent health:

► Which developments have occurred in the health of children and adolescents in Germany over the last ten years?
► Have socioeconomic inequalities in children’s and adolescents’ health widened, narrowed or remained constant?
► How should these inequalities be interpreted in light of the measures and programmes implemented in Germany aimed at promoting child and adolescent health?

## 2. Methodology

### 2.1 Study design

The KiGGS study is part of the health monitoring programme undertaken at the Robert Koch Institute [33]. The following analyses are based on the three cross-sectional surveys conducted as part of the KiGGS study, all of which are representative of 0- to 17-year-old children and adolescents living in Germany. The first cross-sectional survey (KiGGS baseline study) was carried out between 2003 and 2006. It was undertaken as a combined examination and interview survey in a total of 167 communities (sample points) that were representative of Germany’s settlement structure. The addresses of the children and adolescents were selected at random (stratified according to age) from the population registers held by the registry offices in the sample points. In order to attract sufficient numbers of participants with a migration background, the sample was broadened to include more children and adolescents not holding German citizenship. A total of 17,641 children and adolescents (8,656 girls, 8,985 boys) aged between 0 and 17 participated in the study (response rate: 66.6%). In addition to the physical examinations, medical interviews, and various tests and laboratory analyses that were undertaken for the study, parents and participants aged eleven or above were asked to complete a written questionnaire [34].

The second cross-sectional survey (KiGGS Wave 1) was conducted between 2009 and 2012 via telephone interviews. The interviews were based on the questionnaire used for the KiGGS baseline study, but the questions were limited to topics and aspects that could be spoken about in a telephone interview. A total of 12,368 children and adolescents (6,093 girls, 6,275 boys) aged between 0 and 17 took part in the study; parents and participants aged eleven or above were once again provided with a questionnaire. The sample included 7,913 children and adolescents who had already participated in the KiGGS baseline study and who were now aged between 7 and 17 (response rate: 72.9%). In addition, 4,455 children aged between 0 and 6 were newly selected from the population registers from the same sample points and invited to take part (response rate: 38.8%). The aim was to ensure that results from KiGGS Wave 1 were representative of the 0- to 17-year-old population in Germany [35].

The third representative cross-sectional survey (KiGGS Wave 2) was carried out between 2014 and 2017, once again as an examination and interview survey. The survey programme included physical examinations, tests and laboratory analyses as well as a written-postal questionnaire for the parents and for participants aged eleven or above [36]. A new sample stratified according to age was drawn from the registers held by the registry offices in the 167 sample points where the KiGGS baseline study was undertaken. A randomly selected sub-sample of young people aged between 3 and 17 was invited for an examination and an interview; a further sub-sample consisting of children and adolescents aged between 0 and 17 was only invited for an interview. A total of 15,023 children and adolescents (7,538 girls, 7,485 boys) participated in the KiGGS Wave 2 study programme (response rate: 40.1%); 3,567 children and adolescents participated in the additional examination programme (1,801 girls, 1,766 boys) (response rate: 41.5%) [37].

### 2.2 Indicators

This article analyses the following health outcomes: general health, mental health problems, physical activity, the consumption of sugary soft drinks, and smoking. Most of these outcomes focus on children and adolescents aged between 3 and 17. One exception is smoking, where data was collected from children and adolescents aged between 11 and 17. All three surveys provide comparable data for general health, mental health problems and smoking. The analyses of the consumption of sugary soft drinks and physical activity are based on data collected only in two surveys. In the case of physical activity, findings are only based on a period of five (not ten) years.

The analysis of the overall health of children and adolescents is based on data from parental assessments. In the KiGGS study the following question, which is also recommended by the World Health Organization (WHO), was asked: ‘How would you describe the general health of your child?’ (Response categories: ‘Very good’, ‘Good’, ‘Fair’, ‘Poor’, ‘Very poor’) [38]. The responses for ‘Very good’ and ‘Good’, as well as ‘Fair’, ‘Poor’ and ‘Very poor’ were combined to establish two new categories [39].

The data collected on mental health problems as part of the Strengths and Difficulties Questionnaire (SDQ) are also based on information provided by parents [40]. The KiGGS study concentrates on the following four problem areas set out in the questionnaire: emotional symptoms, conduct problems, hyperactivity/inattention and peer relationship problems. 20 statements made by the parents about their children were scored according to the answers provided: ‘Not true’ (0), ‘Somewhat true’ (1) or ‘Certainly true’ (2). The points from each of the areas were then summed. In line with a German standardization [41], children and adolescents with up to 12 points were classified as having no mental health problems, whereas scores of 13 or more points were interpreted as existing mental health problems [42].

In KiGGS Wave 1 and 2, data on physical activity in childhood and adolescence was collected by asking, ‘In a normal week, on how many days are you/is your child physically active for at least 60 minutes per day?’ The answers were provided by parents in the case of children aged between 3 and 10, whereas children and adolescents aged between 11 and 17 were expected to answer the question themselves. The eight response categories ranged from ‘None’ to ‘On seven days’. In the following, a low level of physical activity is assumed for children or adolescents who are physically active for at least 60 minutes per day on less than two days per week [43].

Conclusions about the consumption of sugary soft drinks can be made from data collected with the food frequency questionnaire used for the KiGGS baseline study and KiGGS Wave 2. The questionnaire was filled out by parents of 3- to 10-year-old children and by 11- to 17-year-old children and adolescents themselves [44]. KiGGS Wave 2 posed the following question about sugary soft drinks: ‘How often during the past four weeks did your child/did you drink sugary soft drinks (such as cola, lemonade, ice tea, malt beer or energy drinks)? This does not include diet beverages.’ The following answer categories were provided: ‘Never’, ‘Once per month’, ‘2-3 times per month’, ‘1-2 times per week’, ‘3-4 times per week’, ‘5-6 times per week’, ‘Once per day’, ‘2 times per day’, ‘3 times per day’, ‘4-5 times per day’ and ‘More than 5 times per day’. In addition, data on the mean portion size was collected using the following question: ‘When your child/you drink sugary soft drinks, how much does your child/do you usually drink?’ The answer categories provided were: ‘½ a glass (or less)’, ‘1 glass (200 ml)’, ‘2 glasses’, ‘3 glasses’, ‘4 glasses (or more)’. In the KiGGS baseline study the note ‘This does not include diet beverages’ was not added when asking about the consumption of sugary soft drinks. Instead, data on diet beverages were collected using an additional question. Furthermore, rather than providing the separate answer options ‘2 times per day’ and ‘3 times per day’, the baseline study provided the category ‘2-3 times per day’. In addition, participants were asked to choose from the following portion sizes: ‘¼ a glass (or less)’, ‘½ a glass’, ‘1 glass (200 ml)’, ‘2 glasses’ and ‘3 glasses (or more)’. Estimated mean daily portion sizes were calculated using the information provided about consumption frequency (consumption frequency per 28 days x portion size (g)/28 days). The following analysis distinguishes between children and adolescents who consume less than 500 ml of sugary soft drinks per day and those who consume 500 ml or more per day [45].

In order to collect data on smoking behaviour among adolescents, the KiGGS baseline study and KiGGS Wave 2 asked the written question: ‘Do you currently smoke?’ The following answer categories were provided: ‘No’, ‘Daily’, ‘Several times a week’, ‘Once a week’ and ‘Less [than once a week]’. KiGGS Wave 1 began by asking participants ‘Have you ever smoked?’ (Answer categories were ‘Yes’ and ‘No’.) If this question was answered affirmatively, it was followed by ‘How often do you currently smoke?’ The answer categories provided were very similar to those used in the other two survey waves: ‘Daily’, ‘Several times a week’, ‘Once a week’, ‘Less than once a week’ and ‘Not at all’. In this article, adolescents who used tobacco at all, including only occasionally, are referred to as current smokers [46].

In the following section, social differences in the health of children and adolescents (also referred to hereafter as ‘health inequalities’) are analysed in terms of the socioeconomic status (SES) of the family. SES was calculated consistently across the three survey waves using information provided by parents about their education, occupational qualifications, occupational status, and needs-weighted net household income. An index developed as a sum of point scores, in which the three indicators were included equally, was used to draw a distribution-based demarcation that established three groups: 20% of the children and adolescents were placed in the low (first quintile), 60% in the medium (second to fourth quintile) and 20% in the high status group (fifth quintile) [47].

**Info box: Calculation and interpretation of the Slope Index of Inequality (SII) and the Relative Index of Inequality (RII)**

The SII and the RII are regression-based measures that take into account the overall distribution of socioeconomic variables and the size of socioeconomic groups [48, 49]. Linear probability models were used to compute the SII and log-binomial models were used to calculate the RII. This involved converting the SES variable into a metric scale ranging from 0 (highest SES) to 1 (lowest SES) by means of a ridit analysis [53]. SES was then included as an independent variable in the regression models [52]. The resulting regression coefficients indicate the SII or RII, depending on the respective model. The models included statistical controls for age, gender and a recent history of migration.

SII is to be interpreted as the difference in prevalence (absolute inequality), whereas RII is the prevalence ratio (relative inequality) between adolescents from families with the lowest SES and those from families with the highest SES. For example, an SII of 0.15 would indicate that a 15 percentage-point prevalence difference exists between people at the very bottom and at the very top of the SES scale. An SII value of 0.00 would signify no difference in prevalence between these individuals. An RII of 2.00, for example, indicates that people at the very bottom of the SES scale are twice as likely to have a particular health outcome compared to those at the very top of the SES scale. An RII value of 1.00, in contrast, would indicate that no differences were identified in risk between these individuals.

### 2.3 Statistical methods

Depending on the indicator used, a differing number of participants had to be excluded from the study as they lacked certain information. Prevalences with 95% confidence intervals (CI) were calculated for each health indicator, stratified according to survey period, gender and SES. Trends over time were analysed using logistic regression models with the respective health indicator as the dependent and the survey year as the independent variable. The survey year was included in the model as a linear term. The extent of health inequalities in relation to family SES was analysed using the Slope Index of Inequality (SII) and the Relative Index of Inequality (RII) [48, 49]. Whereas the SII quantifies the extent of absolute inequality, the RII provides a measure of the degree of relative inequality (see Info box). Moreover, since the results of trend analyses of health inequalities and the conclusions that they lead to can be significantly dependent on whether relative or absolute inequalities are considered, it is important that analyses take both dimensions into account [50-52]. Trends over time in terms of absolute and relative health inequalities were analysed using the interaction term between SES and the survey year.

Weighting factors were used to ensure that the samples reflect the official population statistics of the respective survey period in terms of age, gender, federal state, citizenship and parental education. All analyses were performed using Stata 15.1 (StataCorp LP, College Station, TX) survey procedures and took weighting and cluster design effects into account (using cluster-robust standard error estimation). A statistically significant difference between groups is assumed when p-values were less than 0.05 once weighting and the survey design had been taken into account.

## 3. Results

Table 1 describes the sample compositions in relation to gender, age and socioeconomic status (SES). Table 2 depicts developments in the prevalence of selected indicators over the past ten years. In addition to the total values, the prevalences for girls and boys are shown separately. Table 3 sets out prevalences according to SES. The SII and RII shown in Table 4 demonstrate the extent to which absolute and relative inequalities changed over the survey period. Tables 5 to 8 describe the results on developments in prevalence among the socioeconomic status groups and in terms of absolute and relative inequalities, displayed again separately for girls and boys.

### 3.1 General health

In the years 2003-2006, 7.7% of the 3- to 17-year-old children and adolescents in Germany had fair, poor or very poor general health. Between 2003-2006 and 2014-2017, this proportion decreased to 4.3%. The proportion of boys with fair or poor general health was slightly above the corresponding proportion of girls – both at the beginning and end of the observation period. However, the decline in prevalence occurred in a similar way among girls and boys (Table 2). Moreover, it is striking that significant differences were identified throughout the entire observation period to the detriment of the low compared to the medium and in particular to the high socioeconomic status group (Table 3). Prevalence decreased over time in all three status groups. However, as a percentage – in other words, in relative terms – the decline in prevalence was weaker in the low status group than in the medium and high status groups. As such, relative inequalities have widened for general health, and this has occurred equally among girls and boys. By contrast, no significant change was identified in absolute inequalities during the observation period (Table 4).

### 3.2 Mental health problems

The prevalence of mental health problems has decreased over the past decade from 19.8% to 16.9% among 3- to 17-year-old children and adolescents. This is due to the development in boys. No reduction was identified among girls, who are less affected by mental health problems than boys (Table 2). As in general health, a clear social gradient was identified with the highest prevalence in the low status group and the lowest prevalence in the high status group (Table 3). Despite this, absolute inequalities in the prevalence of mental health problems are still significantly higher than in general health. If girls and boys are grouped together, a decline in the prevalence of mental health problems is observed for all three status groups, although no significant change occurs to either relative or absolute inequalities (Table 4). When viewed by gender, only the decline in prevalence among boys in the medium status group is significant.

### 3.3 Low level of physical activity

In contrast to most of the other indicators considered in this work, the proportion of 3- to 17-year-old children and adolescents who were found to have a low level of physical activity has actually risen. Between 2009-2012 and 2014-2017, the prevalence increased from 6.3% to 9.0%. Girls are more likely to show a low level of physical activity than boys, but there are no differences in time trends by gender (Table 2). In addition, the association between low socioeconomic status and a higher proportion of children and adolescents with a low level of physical activity applies to girls just as much as to boys (Table 3). A reduction in relative inequalities was observed during the observation period, which was five years in the case of physical activity (Table 4). This is due to the development in boys, where the increase in prevalence in the medium and high status group was higher than in the low status group. Relative inequalities have remained constant among girls. No significant changes were found among girls or boys in terms of absolute inequalities during the observation period.

### 3.4 Consumption of sugary soft drinks

The proportion of 3- to 17-year-olds that consume 500ml or more of sugary soft drinks per day decreased significantly between 2003-2006 and 2014-2017 from 19.7% to 10.2%. Boys drink sugary soft drinks more often than girls, but the reduction is similar in both genders (Table 2). The relative inequalities to the detriment of the low status group were already very pronounced in 2003-2006 and have widened again until 2014-2017 (Table 4). This increase is due to the fact that the consumption of sugary soft drinks in the medium and especially in the high status group has fallen even more sharply than in the low status group. This trend was identified among girls and boys. At the same time, however, absolute inequalities have decreased, especially among girls.

### 3.5 Smoking

Smoking has declined sharply: whereas in 2003-2006, 21.6% of 11- to 17-year-olds smoked, until 2014-2017 the proportion dropped to just 7.2%. No significant differences as to prevalence or trends were identified between girls and boys (Table 2). However, social differences were observed with regard to tobacco consumption. Girls and boys from families with a low or medium socioeconomic status smoke more often than their peers from families with a high socioeconomic status (Table 3). Nevertheless, the trend analysis shows that the prevalence decreased significantly in all status groups during the observation period. Relative inequalities among girls have remained constant. Absolute inequalities, on the other hand, are significantly reduced (Table 4), which applies to boys and girls.

## 4. Discussion

The data from the KiGGS study indicates that the health of children and adolescents in Germany has improved over the last ten years. There has been, for example, a reduction in the proportion of adolescents with fair, poor or very poor health [39]. The same applies to the proportion of children and adolescents with mental health problems [42]. Further positive developments are that fewer sugary soft drinks are being consumed and that smoking is declining [45, 46]. However, these results stand in contrast to the findings that the proportion of children and adolescents who are physically active for at least 60 minutes per day on less than two days per week increased over the last five years [43]. The developments in the health outcomes described here are similar among girls and boys; the only exception being mental health problems, where a reduction was only observed among boys. Moreover, although the prevalence of mental health problems is lower in girls than boys, the prevalence did not decrease further during the observation period [42].

The KiGGS data point to significant socioeconomic inequalities in young people’s health. The results for all of the health outcomes considered in this article show that children and adolescents from families with a low socioeconomic status are more likely to face disadvantages than their peers in a better socioeconomic situation. In addition, inequalities often exist between children and adolescents from the medium socioeconomic status group compared to those from the high status group. The KiGGS baseline study [54] and KiGGS Wave 1 [28] also identified inequalities according to the socioeconomic status of the family and these results are confirmed by the latest data from KiGGS Wave 2 [31, 55].

The answer to the question raised at the outset – whether socioeconomic inequalities in the health of children and adolescents have changed over the past ten years – depends on the particular health outcome. Moreover, the answer also depends on whether absolute or relative health inequalities are considered. Relative inequalities have widened in general health and the consumption of sugary soft drinks. This is due to the fact that although positive developments were identified among all socioeconomic status groups, they were more pronounced in the medium and high status group than in the low status group. In contrast, relative inequalities in mental health problems and smoking have remained constant over time, and they have even decreased in the case of physical activity. However, the latter reduction is due to the development among boys: during the five-year period, a significantly higher increase in low levels of physical activity was observed for the medium and high status group than for the low status group [56].

No changes were identified in absolute health inequalities over time for general health, mental health problems or physical activity. The results on sugary soft drinks are interesting because they indicate that absolute inequalities narrowed at the same time as relative inequalities significantly widened. The results on smoking demonstrate a significant decline in absolute inequalities with relative inequalities remaining constant. This is understandable given the sharp decline in smoking among all status groups and its current low prevalence.

### Discussion in relation to the current state of research

A number of studies provide information about developments over time regarding the health of children and adolescents in Germany for some but not all of the indicators examined here. Comparable information is available for subjective health, smoking and the consumption of sugary soft drinks. The international Health Behaviour in School-aged Children (HBSC) study provides, for example, data on the health and health-related behaviour of 11- to 15-year-old school children every four years. According to HBSC data for Germany, the proportion of adolescents that view their overall health as ‘fair’ or ‘poor’ (rather than ‘very good’ or ‘good’) decreased slightly between 2002 and 2010 from 14.8% to 13.0% [57]. The decline in smoking, which is clear from the KiGGS data, is supported by results from representative surveys conducted by the Federal Centre for Health Education (BZgA) [58], the European School Survey Project on Alcohol and Other Drugs (ESPAD) [59] and the HBSC study [60, 61]. The BZgA study found that the proportion of 12- to 17-year-olds who smoke fell from around 22% to around 7% between 2003 and 2016 [58]. On the basis of the HBSC data, analyses also can be made about developments in the consumption of sugary soft drinks. The data shows that the proportion of 11- to 15-year-olds who consume sugary soft drinks every day decreased between 2002 and 2014 in Germany as in many other countries [62].

Only a few other studies have investigated developments of socioeconomic inequalities over time in health and health-related behaviour among children and adolescents in Germany. Trend analyses conducted for the HBSC study show that adolescents with low family affluence and a rather poor financial status are more likely to report their health as ‘fair’ or ‘poor’ for all three study years (2002, 2006 and 2010) than their socially better-off peers [57]. The extent of social inequalities in subjective health remained largely constant for both genders over the observation period from 2002 to 2010. In Germany, most studies have focused on developments of social inequalities over time regarding tobacco consumption among adolescents. The studies consistently show that the proportion of girls and boys who smoke has not only decreased significantly since the beginning of the 2000s, and particularly among socioeconomically better-off population groups, but also that considerably fewer young people smoke than 10 to 15 years ago. This even applies to socioeconomically disadvantaged groups [63]. A recent study that determined social status according to the type of secondary school a participant attended, showed that in various surveys (KiGGS, BZgA representative surveys, HBSC, ESPAD), as a result of the reduction in smoking prevalence, absolute inequalities in smoking-related behaviour have mostly decreased, whereas relative inequalities have tended to remain constant or even increase. Students in middle and lower secondary school tracks still smoke more often than those of the same age in higher ones [61].

The majority of international studies on time trends in socioeconomic inequalities in health and health-related behaviour of children and adolescents also use data from the HBSC study [64-67]. Elgar et al. report trends in health inequalities for five indicators using pooled HBSC data from 34 countries [65]. Their findings on physical activity, mental and physical symptoms, body mass index and general satisfaction with life are based entirely on self-reported data collected from the 11- to 15-year-old participants. Between 2002 and 2010, socioeconomic inequalities in health widened in four out of five areas to the disadvantage of socioeconomically deprived young people; general satisfaction with life was the only area in which the extent of social inequalities – the lower the family affluence, the lower the level of satisfaction with life – decreased. A further trend study that focused on physical activity and diet among 15-year-old girls and boys and included data from the latest HBSC wave (2013/2014), concluded that socioeconomic inequalities in physical activity and fruit and vegetable consumption have remained stable or even increased over time [67]. On the other hand, regular consumption of sweets and soft drinks, both summarised in an index as ‘unhealthy diet’, is less associated with family affluence: in 2013/2014, there were no significant differences in this area of nutritional behaviour between adolescents of various social backgrounds in the majority of the countries covered by the HBSC study. Finally, a recent study that evaluated Danish HBSC data also confirmed the finding that physical inactivity among children and adolescents is more widespread in socially disadvantaged families [66]. However, absolute and relative inequalities in physical inactivity remained largely unchanged between 1991 and 2014.

Although a direct, causal attribution cannot be established, it is important to view and interpret changes in health and in socioeconomic inequalities in the health of young people against the background of the measures of health promotion implemented in recent years aimed at promoting child and adolescent health. Correlations may be identified between particular measures and some of the health outcomes considered here; however, this is not always possible, and sometimes only applies to a very limited extent. This was particularly the case for general health because the decline in the proportion of children and adolescents with fair, poor or very poor health could be associated with a variety of causes and can hardly be attributed to the implementation of one particular measure. However, the association is much clearer for other health outcomes such as smoking. Since 2003, efforts to curb smoking and to protect non-smokers from exposure to passive smoking have been stepped up in Germany; for example, tobacco taxes have been raised significantly, smoking bans have been implemented in public places and the sale and marketing of tobacco products is strictly regulated [63]. Many of these measures are aimed at children and adolescents and are intended to prevent or at least complicate the path to taking up smoking [68]. Given the significant decline in smoking and absolute inequalities among adolescents, it is likely that these measures, which are largely attributable to successful structural prevention, have also reached adolescents from families with low socioeconomic status. With this in mind, it seems even more important to continue, extend and adapt these measures to the new products currently being offered by the tobacco industry [69].

At best, the decline in the consumption of sugary soft drinks could be partially associated with a variety of preventive measures. For example, these include measures that have improved the range of drinks on offer and the attractiveness of drinking water and other unsweetened beverages in schools and day-care centres [70, 71]. However, young people continue to consume large amounts of sugary soft drinks, and, as the KiGGS results show, children and adolescents from the low socioeconomic status group do not benefit equally from these measures. In addition to expanding the range of unsweetened drinks on offer in schools and day-care centres, additional preventive measures are currently being discussed with the aim of securing a sharper decline in the consumption of sugary soft drinks. They include the introduction of a sugary soft drinks tax and restrictions on advertising aimed at children and adolescents [72].

The results on physical activity should not only be considered within the context of the National Recommendations for Physical Activity and Physical Activity Promotion [73], but also in terms of the national health target ‘Growing up healthy’ [74], which also promotes exercise. A large proportion of children and adolescents across all status groups were found to undertake a significantly low level of physical activity and this proportion has increased in recent years [43]. The promotion of physical activity in childhood and adolescence should follow a settings-based approach and include measures to ensure that nurseries, schools and the home environment of children and young people become more exercise-friendly. This also includes health-oriented urban planning, the reduction of dangers linked to road traffic and environmental pollution, the expansion of networks of paths for pedestrians and bicycle lanes, and ensuring that green areas and leisure facilities are designed to be child and youth-friendly [73]. These structural preventive measures would also benefit children from socially disadvantaged families, which are proportionally more often physically inactive or only active to a limited extent.

The measures mentioned in connection with the consumption of sugary soft drinks and low levels of physical activity are also relevant with regard to the prevention of overweight. Measures aimed at promoting a healthy diet also need to be taken into account, and these are also addressed as part of the national health target ‘Growing up healthy’ [74] and the National Action Plan ‘IN FORM – Germany’s National Initiative to Promote Healthy Diets and Physical Activity’ [75]. The promotion of a healthy diet and an active lifestyle can influence habits that are otherwise difficult to change in later life [76, 77]. Therefore, efforts to improve the diets of children and adolescents should begin at an early age and be undertaken in environments that are important to children. In addition to the family, this primarily means educational institutions such as day-care centres and schools.

The decline in the prevalence of mental health problems can also be related to specific health-policy measures. In addition to numerous projects conducted in day-care centres and schools, the increased uptake of early detection examinations for children (called U-Untersuchungen in Germany) [78] may have led to better prevention and, therefore, improved mental health. In addition, improved health care may have also contributed to the decline in the prevalence of mental health problems. During the period covered by the KiGGS baseline study, 70% of children and adolescents who displayed symptoms of a mental health problem did not seek psychiatric-psychotherapeutic treatment [79]. Since then, however, the number of child and adolescent psychiatrists taking part in contracted medical care has almost doubled [80]. This increase in specific measures aimed at children and adolescents was partly due to the statutory minimum rate, which was put in place in 2009 and stipulates that 20% of new medical and psychotherapeutic licenses should be reserved for child and adolescent psychotherapy [81].

### Strengths and limitations

One particular strength of the analyses presented here is that developments and trends in the health of children and adolescents and health inequalities are considered using nationwide representative data. The broad samples enable reliable estimates of prevalences, and SII and RII as a measure of absolute or relative health inequalities, over all three observation periods. No other comparable analysis is available for Germany at this time.

However, this study faces a number of limitations that, for example, arise from the fact that KiGGS Wave 1 was conducted as a telephone interview, and the KiGGS baseline study and KiGGS Wave 2 were undertaken as combined examination and interview surveys. In addition, the instruments used to collect data for some health outcomes changed between survey waves. This means that only KiGGS Wave 1 and KiGGS Wave 2 provide comparable data on physical activity, which, in turn, shortens the respective observation period to five years. Changes were also made to the instruments used to study other health outcomes, and this should be taken into consideration when interpreting the results about certain health outcomes in the context of other studies; these changes could have also potentially influenced the results of the trend analyses. This particularly applies to the consumption of sugary soft drinks, since the questionnaires used for this health outcome were not identical. Furthermore, the aim of this article was to analyse health developments and changes to health inequalities in childhood and adolescence using a number of specific health outcomes, and this was done using one indicator in each case. It seems sensible, therefore, that a next step would entail a more differentiated, in-depth analysis using several indicators at the same time.

The question can be raised as to whether three observation periods within ten years are sufficient to provide reliable conclusions about temporal developments and trends. For some of these issues, a longer observation period and a closer sequencing of the surveys would certainly have been desirable. For example, a longer observation period would be valuable for analyses of changes to health inequalities as such changes can often only be identified after a particular time delay. In addition, repeating surveys at shorter intervals would provide a better basis with which to conduct up-to-date assessments of the impact and success of public health policies. Moreover, shortening the interval between surveys would generate a larger number of data points over time. This would mean that non-linear trends could also be analysed, such as whether a decline in prevalence or a rise in health inequalities has continued to increase or begun to decrease over time. As a maximum of three data points were available, the regression models applied in this study only provide for estimates of linear trends.

Nevertheless, it is important to note that the data that was previously available for Germany meant that it was impossible to make any representative conclusions about developments over time and on trends for many health outcomes. In addition, the results of this study clearly show that analyses of temporal developments and trends over the past ten years provide numerous indications about new and changing challenges. The discussion about the associations between developments in health and health inequalities in childhood and adolescence, on the one hand, and health policy measures, on the other, should be approached with caution. For example, the decline in smoking or in the consumption of sugary soft drinks cannot be directly attributed to the policy measures that have been implemented; therefore, it is impossible to confirm how successful they have actually been. At best, it is possible to suggest a temporal coincidence. Be this as it may, the results point to a particularly promising association with regard to the measures implemented in tobacco prevention and control, and the subsequent decline in smoking – all the more so, since smoking has also declined significantly among adolescents from families with low socioeconomic status.

## Key statements

- Children and adolescents from families with a low socioeconomic status are more likely to have health disadvantages than their peers from families with a higher socioeconomic status.
- The extent of the socioeconomic inequalities in mental health problems that occur in childhood and adolescence has remained largely stable over time.
- A more marked decline in the consumption of sugary soft drinks was identified in percentage terms over the course of time in the high status group than in the low status group. Relative inequalities have increased accordingly.
- The proportion of adolescents who smoke has fallen sharply in all status groups; this has also led to a decline in absolute inequalities.

## Figures and Tables

**Table 1:** Characteristics of the KiGGS study populations Source: KiGGS baseline study (2003-2006), KiGGS Wave 1 (2009-2012), KiGGS Wave 2 (2014-2017)

	KiGGS baseline study (2003-2006)		KiGGS Wave 1 (2009-2012)		KiGGS Wave 2 (2014-2017)			
	IS/ES		IS		IS		ES	
	%	n	%	n	%	n	%	n
**Gender**								
Girls	48.7	7,265	48.7	5,154	48.5	6,810	48.5	1,801
Boys	51.3	7,570	51.3	5,272	51.5	6,758	51.5	1,766
**Age group**								
3-10 Years	49.5	8,023	50.5	5,168	51.3	6,969	51.3	1,796
11-17 Years	50.5	6,812	49.5	5,258	48.7	6,599	48.7	1,771
**Socioeconomic status**								
Low	19.9	2,297	20.7	1,074	20.2	1,671	21.6	532
Medium	60.5	8,745	59.7	6,524	60.5	8,257	59.0	2,113
High	19.6	3,492	19.6	2,753	19.4	3,425	19.4	798
**Total**	**100.0**	**14,835**	**100.0**	**10,426**	**100.0**	**13,568**	**100.0**	**3,567**

IS=interview survey, ES=examination survey, n=absolute frequency in the sample (unweighted), %=relative frequency in the population (weighted)

**Table 2:** Prevalence of health outcomes in 3- to 17-year-olds (smoking among 11- to 17-year-olds) according to gender* Source: KiGGS baseline study (2003-2006), KiGGS Wave 1 (2009-2012), KiGGS Wave 2 (2014-2017)

	KiGGS baseline study (2003-2006)		KiGGS Wave 1 (2009-2012)		KiGGS Wave 2 (2014-2017)		
	%	(95% CI)	%	(95% CI)	%	(95% CI)	*p*-trend
**General health (fair to very poor)**							
Total	7.7	(7.1-8.4)	6.4	(5.7-7.1)	4.3	(3.8-4.9)	<0.001
Girls	7.3	(6.4-8.2)	6.6	(5.6-7.6)	4.0	(3.4-4.7)	<0.001
Boys	8.1	(7.3-9.0)	6.2	(5.2-7.2)	4.6	(3.8-5.5)	<0.001
**Mental health problems**							
Total	19.8	(18.9-20.7)	20.2	(18.9-21.6)	16.9	(15.9-17.9)	<0.001
Girls	15.9	(14.8-16.9)	16.9	(15.2-18.7)	14.5	(13.2-15.9)	0.204
Boys	23.6	(22.3-24.9)	23.4	(21.5-25.4)	19.1	(17.7-20.6)	<0.001
**Low level of physical activity**							
Total	–	–	6.3	(5.5-7.3)	9.0	(8.3-9.8)	<0.001
Girls	–	–	8.0	(6.7-9.5)	11.1	(9.9-12.4)	0.001
Boys	–	–	4.7	(3.8-5.9)	7.0	(6.2-8.0)	0.001
**Consumption of sugary soft drinks**							
Total	19.7	(18.6-20.8)	–	–	10.2	(9.4-11.1)	<0.001
Girls	16.3	(15.2-17.6)	–	–	8.1	(7.1-9.1)	<0.001
Boys	22.8	(21.4-24.4)	–	–	12.3	(11.1-13.5)	<0.001
**Smoking**							
Total	21.6	(20.4-22.9)	12.0	(10.8-13.3)	7.2	(6.3-8.2)	<0.001
Girls	22.0	(20.3-23.7)	11.9	(10.2-13.8)	7.4	(6.2-8.9)	<0.001
Boys	21.3	(19.6-23.1)	12.1	(10.5-14.0)	7.0	(5.9-8.2)	<0.001

* weighted by the population structure in the respective study period, CI=confidence interval

**Table 3:** Prevalence of health outcomes among 3- to 17-year-olds (smoking among 11- to 17-year-olds) according to socioeconomic status* Source: KiGGS baseline study (2003-2006), KiGGS Wave 1 (2009-2012), KiGGS Wave 2 (2014-2017)

	KiGGS baseline study (2003-2006)		KiGGS Wave 1 (2009-2012)		KiGGS Wave 2 (2014-2017)		
	%	(95% CI)	%	(95% CI)	%	(95% CI)	*p*-trend
**General health (fair to very poor)**							
Low SES	11.4	(9.7-13.4)	10.6	(8.3-13.6)	7.7	(6.1-9.6)	0.003
Medium SES	7.5	(6.8-8.3)	5.9	(5.1-6.7)	4.1	(3.5-4.6)	<0.001
High SES	4.4	(3.7-5.3)	3.2	(2.5-4.1)	1.4	(1.0-1.9)	<0.001
**Mental health problems**							
Low SES	30.6	(28.3-33.1)	33.5	(29.6-37.6)	26.0	(23.3-28.9)	0.031
Medium SES	19.0	(17.9-20.1)	19.0	(17.5-20.6)	16.1	(15.0-17.4)	0.002
High SES	11.2	(10.3-12.2)	9.8	(8.6-11.3)	9.7	(8.7-10.8)	0.028
**Low level of physical activity**							
Low SES	–	–	11.9	(9.2-15.3)	15.4	(12.9-18.2)	0.094
Medium SES	–	–	5.8	(5.1-6.7)	7.9	(7.1-8.8)	<0.001
High SES	–	–	2.3	(1.7-3.1)	5.9	(5.0-6.9)	<0.001
**Consumption of sugary soft drinks**							
Low SES	28.9	(26.4-31.5)	–	–	17.9	(15.7-20.3)	<0.001
Medium SES	20.3	(19.0-21.6)	–	–	10.3	(9.3-11.4)	<0.001
High SES	9.0	(7.9-10.3)	–	–	2.6	(1.9-3.4)	<0.001
**Smoking**							
Low SES	25.2	(22.4-28.3)	14.4	(11.1-18.5)	8.0	(5.6-11.4)	<0.001
Medium SES	21.5	(19.8-23.2)	11.8	(10.4-13.4)	7.9	(6.8-9.2)	<0.001
High SES	16.3	(14.2-18.7)	8.9	(7.1-11.1)	4.0	(2.8-5.6)	<0.001

* weighted by the population structure in the respective study period, SES=socioeconomic status, CI=confidence interval

**Table 4:** Absolute and relative inequalities (SII and RII) of different health outcomes among 3- to 17-year-olds (smoking among 11- to 17-year-olds)* Source: KiGGS baseline study (2003-2006), KiGGS Wave 1 (2009-2012), KiGGS Wave 2 (2014-2017)

	KiGGS baseline study (2003-2006)		KiGGS Wave 1 (2009-2012)		KiGGS Wave 2 (2014-2017)		
	(95% CI)		(95% CI)		(95% CI)		*p*-trend
**General health (fair to very poor)**							
SII	0.06	(0.04-0.09)	0.07	(0.04-0.11)	0.07	(0.05-0.10)	0.399
RII	2.26	(1.64-3.12)	3.26	(1.88-5.66)	6.04	(3.81-9.58)	0.001
**Mental health problems**							
SII	0.22	(0.19-0.26)	0.28	(0.23-0.34)	0.21	(0.17-0.25)	0.899
RII	3.11	(2.62-3.67)	4.15	(3.19-5.39)	3.63	(2.90-4.54)	0.128
**Low level of physical activity**							
SII	–	–	0.09	(0.06-0.13)	0.09	(0.06-0.12)	0.907
RII	–	–	4.21	(2.60-6.82)	2.95	(2.10-4.12)	0.215
**Consumption of sugary soft drinks**							
SII	0.25	(0.22-0.29)	–	–	0.20	(0.17-0.23)	0.009
RII	3.35	(2.86-3.94)	–	–	6.78	(5.04-9.10)	<0.001
**Smoking**							
SII	0.16	(0.12-0.20)	0.07	(0.01-0.12)	0.04	(0.004-0.08)	<0.001
RII	2.04	(1.70-2.47)	1.58	(1.05-2.37)	1.78	(1.06-2.99)	0.388

SII=Slope Index of Inequality, RII=Relative Index of Inequality, CI=confidence interval

* adjusted for age, gender, age × gender and migration background

**Table 5:** Trends in the prevalence of health outcomes for 3- to 17-year-old girls (smoking among 11- to 17-year-olds) according to socioeconomic status* Source: KiGGS baseline study (2003-2006), KiGGS Wave 1 (2009-2012), KiGGS Wave 2 (2014-2017)

	KiGGS baseline study (2003-2006)		KiGGS Wave 1 (2009-2012)		KiGGS Wave 2 (2014-2017)		
	%	(95% CI)	%	(95% CI)	%	(95% CI)	*p*-trend
**General health (fair to very poor)**							
Low SES	11.0	(8.8-13.7)	10.0	(6.9-14.3)	6.8	(5.1-9.0)	0.004
Medium SES	6.8	(5.9-7.9)	6.5	(5.4-7.8)	3.9	(3.2-4.6)	<0.001
High SES	4.7	(3.6-6.1)	3.2	(2.3-4.4)	1.0	(0.6-1.7)	<0.001
**Mental health problems**							
Low SES	26.5	(23.5-29.9)	29.4	(23.9-35.6)	22.7	(19.3-26.4)	0.157
Medium SES	14.7	(13.4-16.2)	15.7	(14.0-17.7)	14.3	(12.8-16.0)	0.816
High SES	8.3	(7.0-9.8)	8.0	(6.6-9.6)	6.4	(5.2-7.9)	0.117
**Low level of physical activity**							
Low SES	–	–	13.1	(9.4-18.1)	19.4	(15.8-23.6)	0.040
Medium SES	–	–	8.0	(6.7-9.4)	9.6	(8.3-11.1)	0.093
High SES	–	–	3.3	(2.3-4.7)	7.6	(6.2-9.4)	<0.001
**Consumption of sugary soft drinks**							
Low SES	25.1	(21.9-28.6)	–	–	13.5	(11.0-16.5)	<0.001
Medium SES	16.9	(15.5-18.4)	–	–	8.4	(7.2-9.9)	<0.001
High SES	6.2	(4.9-7.9)	–	–	1.5	(1.0-2.3)	<0.001
**Smoking**							
Low SES	27.2	(22.8-32.0)	13.9	(9.2-20.5)	9.2	(6.0-13.9)	<0.001
Medium SES	21.9	(19.6-24.3)	12.3	(10.1-15.0)	7.6	(6.2-9.4)	<0.001
High SES	15.2	(12.8-18.0)	7.5	(5.2-10.5)	4.3	(2.6-7.0)	<0.001

* weighted by the population structure in the respective study period, SES=socioeconomic status, CI=confidence interval

**Table 6:** Trends in the prevalence of health outcomes for 3- to 17-year-old boys (smoking among 11- to 17-year-olds) according to socioeconomic status* Source: KiGGS baseline study (2003-2006), KiGGS Wave 1 (2009-2012), KiGGS Wave 2 (2014-2017)

	KiGGS baseline study (2003-2006)		KiGGS Wave 1 (2009-2012)		KiGGS Wave 2 (2014-2017)		
	%	(95% CI)	%	(95% CI)	%	(95% CI)	*p*-trend
**General health (fair to very poor)**							
Low SES	11.8	(9.6-14.5)	11.2	(8.1-15.2)	8.5	(6.2-11.6)	0.092
Medium SES	8.1	(7.1-9.2)	5.2	(4.3-6.4)	4.2	(3.4-5.2)	<0.001
High SES	4.1	(3.3-5.2)	3.2	(2.4-4.4)	1.6	(1.0-2.5)	<0.001
**Mental health problems**							
Low SES	34.5	(31.0-38.2)	37.0	(31.2-43.3)	29.0	(24.8-33.7)	0.094
Medium SES	23.1	(21.5-24.7)	22.1	(20.1-24.3)	17.9	(16.1-19.8)	<0.001
High SES	14.0	(12.6-15.6)	11.6	(9.6-14.0)	12.7	(10.9-14.7)	0.193
**Low level of physical activity**							
Low SES	–	–	10.9	(7.4-15.7)	11.6	(8.6-15.5)	0.791
Medium SES	–	–	3.7	(3.0-4.7)	6.3	(5.3-7.4)	0.001
High SES	–	–	1.3	(0.8-2.1)	4.4	(3.3-5.8)	<0.001
**Consumption of sugary soft drinks**							
Low SES	32.5	(28.7-36.4)	–	–	21.9	(18.5-25.8)	<0.001
Medium SES	23.5	(21.8-25.3)	–	–	12.2	(10.8-13.7)	<0.001
High SES	11.7	(9.9-13.7)	–	–	3.5	(2.5-4.8)	<0.001
**Smoking**							
Low SES	23.2	(19.0-28.1)	14.8	(10.2-20.9)	6.7	(4.2-10.4)	<0.001
Medium SES	21.1	(19.0-23.4)	11.3	(9.6-13.3)	8.2	(6.7-10.1)	<0.001
High SES	17.4	(14.3-21.1)	10.3	(7.9-13.2)	3.7	(2.3-5.9)	<0.001

* weighted by the population structure in the respective study period, SES=socioeconomic status, CI=confidence interval

**Table 7:** Trends in absolute and relative inequalities (SII and RII) for different health outcomes among 3- to 17-year-old girls (smoking among 11- to 17-year-olds)* Source: KiGGS baseline study (2003-2006), KiGGS Wave 1 (2009-2012), KiGGS Wave 2 (2014-2017)

	KiGGS baseline study (2003-2006)		KiGGS Wave 1 (2009-2012)		KiGGS Wave 2 (2014-2017)		
	(95% CI)		(95% CI)		(95% CI)		*p*-trend
**General health (fair to very poor)**							
SII	0.05	(0.02-0.09)	0.07	(0.02-0.13)	0.07	(0.04-0.10)	0.426
RII	2.17	(1.31-3.61)	3.10	(1.42-6.76)	6.13	(3.43-10.94)	0.010
**Mental health problems**							
SII	0.21	(0.16-0.25)	0.26	(0.18-0.33)	0.21	(0.16-0.26)	0.883
RII	3.79	(2.86-5.01)	4.70	(3.10-7.13)	4.22	(3.07-5.80)	0.525
**Low level of physical activity**							
SII	–	–	0.08	(0.03-0.12)	0.11	(0.06-0.16)	0.373
RII	–	–	2.53	(1.49-4.29)	2.67	(1.69-4.22)	0.871
**Consumption of sugary soft drinks**							
SII	0.24	(0.20-0.28)	–	–	0.15	(0.12-0.19)	0.002
RII	4.19	(3.26-5.38)	–	–	7.04	(4.44-11.16)	0.039
**Smoking**							
SII	0.20	(0.13-0.26)	0.07	(-0.01-0.15)	0.05	(-0.002-0.11)	<0.001
RII	2.47	(1.88-3.26)	1.85	(1.003-3.40)	2.03	(0.95-4.33)	0.372

SII=Slope Index of Inequality, RII=Relative Index of Inequality, CI=confidence interval

* adjusted for age and migration background

**Table 8:** Trends in absolute and relative inequalities (SII and RII) for different health outcomes among 3- to 17-year-old boys (smoking among 11-17 year-olds)* Source: KiGGS baseline study (2003-2006), KiGGS Wave 1 (2009-2012), KiGGS Wave 2 (2014-2017)

	KiGGS baseline study (2003-2006)		KiGGS Wave 1 (2009-2012)		KiGGS Wave 2 (2014-2017)		
	(95% CI)		(95% CI)		(95% CI)		*p*-trend
**General health (fair to very poor)**							
SII	0.07	(0.04-0.10)	0.07	(0.03-0.12)	0.08	(0.05-0.11)	0.657
RII	2.34	(1.62-3.39)	3.46	(1.70-7.04)	5.93	(3.12-11.26)	0.013
**Mental health problems**							
SII	0.24	(0.19-0.29)	0.31	(0.22-0.40)	0.22	(0.16-0.28)	0.735
RII	2.78	(2.22-3.48)	3.84	(2.67-5.53)	3.30	(2.43-4.47)	0.231
**Low level of physical activity**							
SII	–	–	0.10	(0.05-0.16)	0.08	(0.04-0.13)	0.549
RII	–	–	10.35	(4.14-25.84)	3.43	(1.93-6.12)	0.058
**Consumption of sugary soft drinks**							
SII	0.27	(0.22-0.32)	–	–	0.24	(0.20-0.29)	0.436
RII	2.96	(2.39-3.66)	–	–	6.68	(4.67-9.57)	<0.001
**Smoking**							
SII	0.12	(0.06-0.19)	0.06	(-0.02-0.14)	0.03	(-0.01-0.08)	0.021
RII	1.70	(1.28-2.25)	1.40	(0.78-2.51)	1.61	(0.86-3.01)	0.679

SII=Slope Index of Inequality, RII=Relative Index of Inequality, CI=confidence interval

* adjusted for age and migration background
